# Urodynamic study findings and related influential factors in pediatric spastic cerebral palsy

**DOI:** 10.1038/s41598-022-11057-3

**Published:** 2022-04-28

**Authors:** Wenbin Jiang, Huizhen Sun, Baojun Gu, Qijia Zhan, Min Wei, Sen Li, Fang Chen, Bo Xiao

**Affiliations:** 1 Department of Neurosurgery, Shanghai Children’s Hospital, Shanghai Jiao Tong University, 355 Luding Road, Shanghai, 200062 China; 2 Department of Urology, Shanghai Children’s Hospital, Shanghai Jiao Tong University, Shanghai, 200062 China; 3 Department of Urology, Shanghai Jiao Tong University Affiliated Sixth People’s Hospital, Shanghai, 200233 China

**Keywords:** Bladder, Paediatric urology

## Abstract

To investigate the urodynamic study (UDS) result in pediatric patients suffering from spastic cerebral palsy (CP). Medical records of patients diagnosed CP having pre-operative UDS results underwent selective dorsal rhizotomy (SDR) from Jan. 2020 to May. 2021 were retrospectively reviewed. Fifty-seven cases diagnosed spastic CP were included in the study (mean age, 6.73 ± 2.84 years), among which, 46 were ambulatory and 11 non-ambulatory. Average gross motor function measure-66 (GMFM-66) score was 62.16 ± 11.39. Reduced bladder capacity was seen in 49.12% of these cases and cases with lower GMFM-66 score possessed a higher incidence rate of having low bladder capacity (*p* < 0.01). Detrusor overactivity (DO) was shown in 33.33% of the patients. Cases with younger age presented a higher prevalence of DO (*p* < 0.05). Meanwhile, more non-ambulant patients suffered from DO (*p* < 0.05). Increased post-voiding residual (PVR) was seen in 21.05% of the cases. Those with higher average threshold in sphincter-associated input spinal nerve roots (rootlets) witnessed a higher rate of having abnormal PVR (*p* < 0.05). Abnormal UDS results were prevalent in pediatric patients suffering from CP. Motor function, age and threshold of their sphincter-associated spinal nerve roots laid corresponding effect on the abnormal UDS results.

## Introduction

Urodynamic study (UDS), known as an effective quantitative measure to assess urinary function, has been clinically applied for many years^1^. Micturition dysfunctions are commonly seen in both adults and children suffering from spastic cerebral palsy (CP), and it is reported that 30% children with CP suffers from urinary incontinence and neurogenic voiding dysfunction^2,3^. Previous emphasis were generally placed on the impact of spasticity on the motor function development of these patients while most existing researches applying UDS/uroflowmetry were restricted by their sample size, making it difficult to conduct cohorts analysis^4,5^. Much fewer literature investigated micturition function via UDS in children with CP in the past 5 years.

Single-level approach selective dorsal rhizotomy (SDR), a neurosurgical operation to lower spasticity in patients with CP^6^, requires the operators to identify each root (rootlet) of cauda equina at L2 level via trigger electromyography (EMG) interpretation to figure out sensory roots (rootlets) associated with lower limb spasticity. Sacral sensory roots (rootlets) were highly associated with the bladder/bowel function and should be carefully protected intraoperatively in case of the occurrence of post-operational urinary dysfunction^7^. SDR procedure provides an effective way of recording electro-neurophysiological data of each root (rootlet) in caudal equina during surgeries. The combination of these data with pre-operational UDS results in patients with spastic CP enables researchers to learn more about the underlying mechanisms of urinary disorder.

This study was therefore carried out to review UDS findings in pediatric patients suffering from spastic CP who had undergone SDR procedure in the center, intended to grasp enriched understandings of the characteristics of abnormal bladder function, as well as their neuro-electrophysiology of sacral sensory roots in this population.

## Material and methods

Medical records of pediatric patients diagnosed spastic CP operated on using SDR at the Center from Jan. 2020 to May. 2021 were retrospectively reviewed. The indications for SDR were previously documented in the paper ever published^6^. Pre-op UDS examination was carried out for all included cases before SDR. The diagnosis of spastic CP was conducted by a multi-disciplinary team consisting of neurosurgeons, neurologists and physical therapists. Clinical data including demographics, urodynamic results, relevant evaluation records, and intra-operative neurophysiological data of the included cases were adopted from the Database of Pediatric Cerebral Palsy Patients of the hospital.

### Motor function assessment

Gross motor function classification system (GMFCS), a five-level classification system depicting functional abilities in sitting, walking and need for assistive devices such as walkers or wheelchairs, and gross motor function measure-66 (GMFM-66), a standardized observational instrument designed to measure change in gross motor function over time^9^, were applied to assess the motor function of the cases. In GMFCS, Children in Level I can generally walk without restrictions but tend to be limited in some more advanced motor skills while those in Level V are usually limited in their ability to move around^8^.

### UDS

The UDS test was conducted in accordance with the standardization of the International Children’s Continence Society (ICCS)^10^. All tests were carried out with Ellipse (ANDROMEDA MedizinischeSysteme GmbH, Wallbergstr. 5, 82024 Taufkirchen/Potzham Germany). UDS was performed with slow fill water cystometry with a 6-Fr double lumen UDS catheter, with patient keeping supine without sedation and a rectal catheter was inserted into the anal to measure the abdominal pressure. The infusion rate of saline was 10% estimated bladder capacity (EBC) per minute, and age-related bladder capacity was calculated via the equation (age + 1) × 30 ml in patients younger than 11 years old and a minimum capacity of 350 ml was set as the baseline for patients aged 12 years or older^11^. Saline filling was discontinued when the child indicated fullness or when substantial leakage occurred. Clinical test was followed by kidneys sonography when the bladder was empty and residual urine was measured twice. The lowest residual urine measurement was adopted for the evaluation.

**Figure 1:**
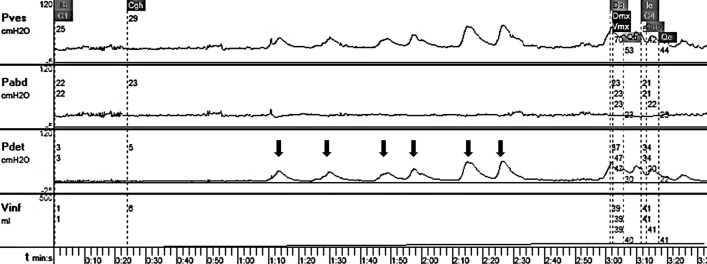
Representative urodynamic result of spastic cerebral palsy showing involuntary detrusor overactivity during filling phase. *Pdet* Pves–Pabd. *X-axis* time, *Y-axis* pressure (cm H_2_O). Arrows: non-voiding contractions.

Total bladder capacity was defined as the total volume of water infused into the bladder until the filling end point while small bladder referred to a bladder whose capacity was less than 65% EBC^12^. Involuntary detrusor pressure increase over 15 cm H2O was considered as an overactive detrusor contraction (Fig. 1)^13^. Post-voiding residual (PVR) ≥ 20 ml was defined as the elevated PVR in accordance with the definition made by ICCS^14^. Other UDS parameters, such as bladder compliance measured with algorithm ΔV/ΔP_det_ (ΔV refers to the mean volumetric capacity; ΔP_det_, the detrusor pressure rise) were also included. Children with a bladder compliance of 15 mL/cm H_2_O or higher were defined as normal^15^.

### Intra-operative neurophysiology

**Figure 2:**
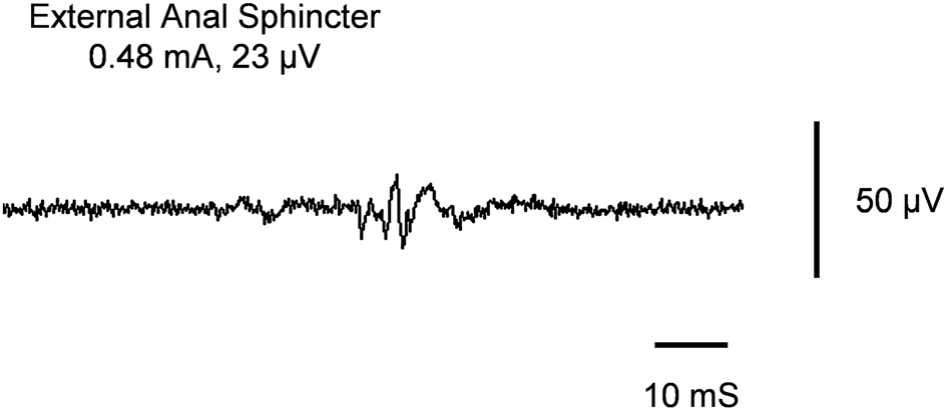
Intraoperative trigger electromyography during the surgery. The freezing screen presented a representative external anal sphincter activity elicited by electrically stimulating a dorsal rootlet. This stimulated rootlet is defined as sphincter-associated input rootlet (SAIR).

All patients underwent SDR guided by the modified intra-operative neurophysiologic protocol^16^. Surgeries were performed under general anesthesia with the minimum alveolar concentration (MAC) of sevoflurane inhalation at 0.5 and maintenance of body temperature between 36.0 and 37.0 °C. A total of 15 channels, including external anal sphincter (EAS), were neuro-physiologically monitored, with all spinal nerve roots (rootlets) at L2 level stimulated. The monitoring system adopted by the Center was Cadwell-Cascade Elite. In accordance with the protocol, all sensory roots (rootlets) on which single pulse stimulation first evoked responses in EAS channel were defined as sphincter-associated input roots (rootlets) (SAIR). The electrical current given to SAIR, which elicited responses reaching around 20 μV in EAS channel, was set as its threshold (Fig. 2). Given the fact that under normal circumstances, 10–20 roots (rootlets) could match the definition of SAIR based on the protocol in each case, an average of electrical threshold in all these rootlets in each case became the focus to be further discussed.

### Statistical analysis

Continuous variables with normal distribution were presented as mean ± SD and non-normal variables were reported as median (Q1, Q3). Continuous variables were compared via the *t* test or the non-parametric Mann–Whitney *U* test whenever appropriate and statistical comparison was conducted via the Chi-square test for categorical data, with continuity correction adopted whenever appropriate. Receiver-operating characteristic (ROC) curves were generated for the analysis of factors affecting abnormal UDS results. The value of *p* < 0.05 was considered statistically significant. The data were analyzed by SPSS version 24.0 for Windows (SPSS Inc., Chicago, IL, USA).

### Ethical approval

This study was conducted in accordance with the relevant guidelines and the Declaration of Helsinki. It is a retrospective study of clinical data and it has been approved by the Ethics Review Committee, Children’s Hospital of Shanghai, Shanghai Jiao Tong University (Approval No: 2020R069-E02). Because of the retrospective nature of the study, the informed consent for inclusion was waived by the ethics committee of Children’s Hospital of Shanghai.

## Results

A total of 57 cases were observed, among which, 44 (77.19%) were boys. The mean age at the time of SDR was 6.73 ± 2.84 within the range from 3 to 17 years old. The most two common etiologies of CP were asphyxia (56.14%) and premature (24.56%). In cases included, 8 (14.04%), 39 (68.42%) and 10 (17.54%) were spastic hemiplegic, diplegic and quadriplegic, respectively. Among the cases, 46 (80.70%) were ambulatory (GMFCS level I to III) whilst 11 (19.30%) were non-ambulatory (level IV to V). The mean pre-op GMFM-66 score was 62.16 ± 11.39 in these cases within the range from 29.96 to 82.99. Detailed demographic information was shown in Table 1.

**Table 1:** Demographic details of 57 cases included in this study.

Characteristics	No., %
**Gender**	
Boy	44, 77.19%
Girl	13, 22.81%
**Age at surgery, year (mean ± SD)**	6.73 ± 2.84
**Etiology of spasticity**	
Premature	14, 24.56%
Asphyxia	32, 56.14%
Unknown	11, 19.30%
**Spastic type**	
Hemiplegia	8, 14.04%
Diplegia	39, 68.42%
Quadriplegia	10, 17.54%
**GMFCS level**	
Level I	4, 7.02%
Level II	20, 35.09%
Level III	22, 38.60%
Level IV	10, 17.54%
Level V	1, 1.75%
GMFM-66 score (mean ± SD)	62.16 ± 11.39
**Bladder/bowel symptoms**	
Enuresis	4, 7.02%
Urge urinary incontinence	17, 29.82%
Frequency	7, 12.28%
Urgency	5, 8.77%
Normal voiding	27, 47.37%
Constipation	17, 29.82%
Normal defecation	40, 70.17%
**Urodynamic outcomes**	
Overactive Bladder	19, 33.33%
Small bladder capacity	28, 49.12%
Increased post-voiding residual	12, 21.05%
Normal	17, 29.82%

*GMFCS* gross motor function classification system, *GMFM-66* gross motor function measurement-66.

In accordance with their previous experiences offered by parents, all children could voluntarily control their bladder and bowel, except a 3-year-old boy requiring constant diaper-wearing. Thirty patients (52.63%) presented one or more lower urinary tract symptoms (LUTS), among which, 25 showed abnormal UDS performance, while in other 27 cases not presenting LUTS, 15 (26.32%) presented abnormal UDS results (*p* < 0.05). Intermittent daytime urinary incontinence was the most common symptom presented in 17 cases (29.82%), among which, 14 had daytime incontinence nearly once a week, whilst the others, once a day; 6 (35.29%) suffered from DO, 12 (70.59%), reduced bladder capacity and 5 (29.41%), increased PVR. Frequency (> 7 times per day), urgency and enuresis were observed in 7 (12.28%), 5 (8.77%) and 4 (7.02%) cases, respectively. Among 7 children with increased voiding frequency, 6 (85.71%) had a small bladder capacity, 3 (42.86%) were found with bladder overactivity and only one (14.29%) had increased PVR. In addition to LUTS, 17 (29.82%) cases suffered from bowel symptoms (constipation).

**Figure 3:**
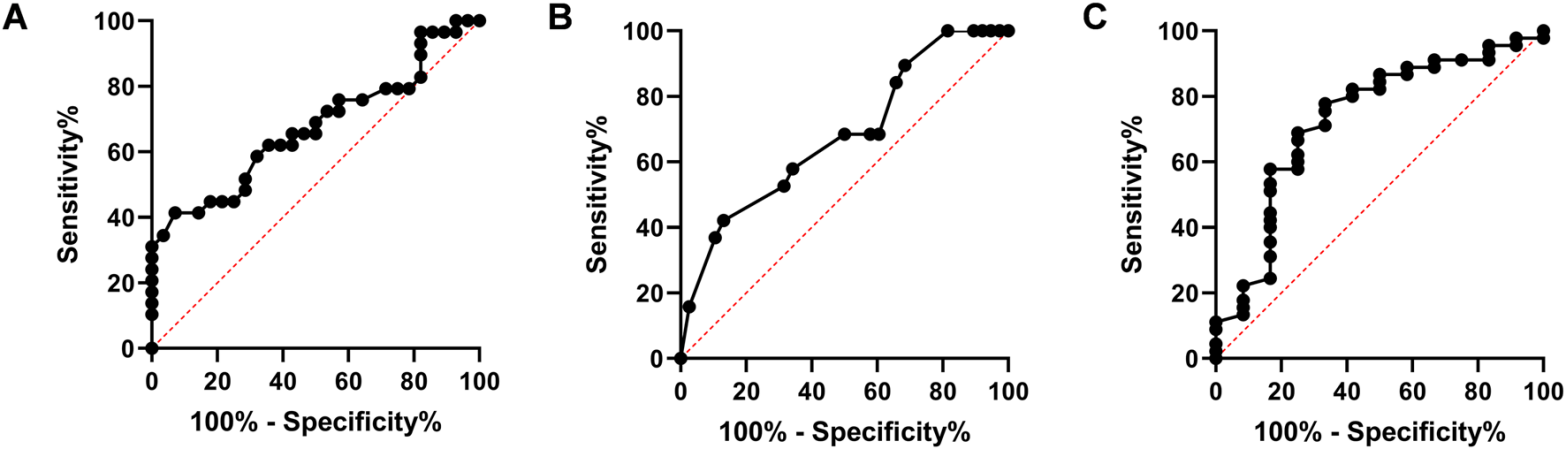
(**A**) ROC curve of GMFM-66 scores in cases with reduced bladder capacity (AUC: 0.67; CI 0.53–0.81; *p* = 0.03; cut-off point: 70.22, sensitivity: 41.38%, specificity: 92.86%). (**B**) ROC curve of age in cases with DO (AUC: 0.68; CI 0.53–0.83; *p* = 0.03; cut-off point: 4.75 years old, sensitivity: 42.11%, specificity: 86.84%). (**C**) ROC curve of SAIR threshold in cases with elevated PVR (AUC: 0.73; CI 0.56–0.90; *p* = 0.02; cut-off point: 0.72 mA, sensitivity: 77.78%, specificity: 66.67%). *ROC* receiver operating characteristic, *GMFM-66* gross motor function measurement-66, *AUC* area under curve, *DO* detrusor overactivity, *SAIR* sphincter-associated input roots (rootlets), *PVR* post-voiding residual.

Abnormal UDS findings were revealed in 40 cases (70.18%). The bladder capacity of the 57 cases ranged from 55 to 450 ml, and the average figure was 60.63% of the estimated bladder capacity (EBC), Twenty-eight (49.12%) from the 57 cases had a bladder capacity less than 65% EBC. Detailed statistical comparisons were listed in Table 2. Compared to cases with a normal/small bladder capacity, significant difference was also found in the GMFM-66 score. It was observed that cases with a GMFM-66 score less than 70.22 were more likely to have a small bladder capacity. The cut-off point (70.22) was calculated with receiver operating characteristic (ROC) curve, with an area under curve (AUC) equaling 0.67 (Fig. 3A, *p* < 0.05).

**Table 2:** Comparisons between cases with and without UDS abnormalities.

Characteristics	Bladder capacity			Detrusor activity			PVR		
	Decreased	Normal	*p* value	Overactivity	Normal	*p* value	Increased	Normal	*p* value
**Categorical data**									
Gender (n)									
Boy	20	24	0.31	16	28	0.58*	9	35	0.85 *
Girl	8	5		3	10		3	10	
GMFCS (n)									
Level I–III	21	25	0.46*	12	34	0.04*	9	37	0.88*
Level IV–V	7	4		7	4		3	8	
**Quantitative data**									
Age (years)									
Median (Q1, Q3)	6.50 (5.00, 8.50)	5.00 (4.00, 9.00)	0.26	5.00 (4.00, 8.00)	6.25 (5.00, 9.00)	0.03	6.50 (5.50, 10.75)	6.00 (4.50, 8.50)	0.12
95% CI	5.50–8.00	4.50–6.50		4.00–8.00	5.50–8.50		5.50–11.50	5.00–8.00	
GMFM-66 score									
Mean ± SD	58.20 ± 10.27	65.98 ± 11.46	< 0.01	59.95 ± 13.27	63.26 ± 10.50	0.31	59.46 ± 8.97	62.88 ± 12.05	0.36
95% CI	54.22–62.18	61.62–70.34		53.56–66.35	59.81–66.71		53.76–65.16	59.26–66.50	
Number of SAIR (n)									
Mean ± SD	16.71 ± 5.72	17.55 ± 5.20	0.56	18.21 ± 7.12	16.61 ± 4.36	0.30	16.58 ± 5.85	17.29 ± 5.37	0.69
95% CI	14.50–18.93	15.57–19.53		14.78–21.64	15.17–18.04		12.86–20.30	15.68–18.90	
Average threshold of SAIR (mA)									
Median (Q1, Q3)	0.47 (0.37, 0.72)	0.54 (0.33, 1.09)	0.63	0.47 (0.38, 1.07)	0.51 (0.32, 0.75)	0.46	0.86 (0.52, 1.42)	0.46 (0.35, 0.67)	0.01
95% CI	0.38–0.64	0.36–0.80		0.38–1.07	0.38–0.64		0.50–1.43	0.38—0.55	

*PVR* post-voiding residual, *GMFCS* gross motor function classification system, *GMFM-66* gross motor function measurement-66, *SAIR* sphincter-associated input roots (rootlets).

*Continuity correction.

DO was observed in 19 (33.33%) cases with their involuntary detrusor pressure measured over 15 cm H_2_O in the filling phase during the UDS test. It turned out that non-ambulatory cases had more chance of suffering from DO than those ambulatory ones (Table 2, *p* < 0.05). Statistical analysis showed that patients younger than 4.75 years old tended to have more chance of suffering from DO (cut-off point got from the ROC curve with an AUC equaling 0.68, Fig. 3B, *p* < 0.05).

Increased PVR was noticed in 12 (21.05%) cases with their PVR ranging from 20 to 139 ml. The rest 45 patients had a PVR less than 10 ml. Corresponding statistical comparisons were listed in Table 2. Intra-operative neurophysiological data showed that median threshold of SAIR was 0.5 mA (Q1, Q3: 0.36, 0.78 mA) in the 57 cases, much greater in cases with increased PVR than those with normal residual (median: 0.86 vs. 0.46 mA, *p* < 0.01). As demonstrated by the data, cases in which average threshold of SAIR over 0.72 mA were more likely to be found with increased residual after voiding (cut-off value got from ROC curve with AUC equaling 0.73, Fig. 3C, *p* < 0.05).

Bladder compliance with a mean of 29.29 ± 12.95 ml/cm H_2_O (ranging from 15.00 to 65.67 ml/cm H_2_O) was observed in all the cases, all of which turned out to be within the normal range.

## Discussion

It has been reported that patients suffering from spastic CP commonly presented dysfunctional urinary symptoms, such as urge urinary incontinence, frequency and urgency. In accordance with published papers^17–19^, incident rate of LUTS in CP ranged from 40.74% to 93.94% while the prevalence of LUTS in the 57 cases was 52.63% (30 cases), among which, 18 were proven to reveal multiple presentations. The results were similar to those acquired in previous studies. In these 30 cases with LUTS, 25 of them presented abnormal UDS performance. Even in other 27 cases without LUTS, 15 of them revealed abnormal results in UDS. The results perfectly tallied with previous studies wherein cases suffering from CP might exhibit silent bladder dysfunction even when found without LUTS^20^. Additionally, in cases suffering from CP presented with LUTS, the UDS results were more likely to be abnormal. The essential role played by UDS in detecting the potential voiding dysfunction in patients with spastic CP was further highlighted through these findings.

The most common UDS result in patients suffering from spastic CP was proven to be a decreased bladder capacity^3,21^. In the study, nearly 50% of the cases was found to have a bladder capacity less than 65% EBC, comparable with previous studies^22^. It is speculated that parents reduced the water intake of the children in this group to lower the burden of daily care^23^. The presence of overactive bladder was not an uncommon finding in this population^4,19^. UDS in the cohort had verified the similar result that DO occurred in 19 cases (33.33%). It is therefore further speculated that DO might be resulted from lack of inhibition from upper motor neurons, leading to the overactivity of lower motor neurons^3^. Interestingly, it is also found that 11 out of 19 (57.89%) cases with DO also have a decreased bladder capacity, making it a reasonable assumption that DO might impact the bladder distention in an evolved bladder in the childhood of these patients, eventually causing a reduced bladder capacity. Statistical comparison showed that cases with more impaired motor function were more likely to have DO and a reduced bladder capacity than those with less. This might be attributed to more severe brain damage. In addition, data showed that younger children tended to be more likely to have DO than older ones. Possible explanation was the immaturity of a developing nervous system^24^.

The value of bladder compliance varies with age, and can be affected by the rate of bladder filling, making it a rather complicated issue in pediatric patients. The study chose the cut-off point of 15 ml/cmH_2_O and an overall normal compliance was found. The data were in line with findings previously documented^12,19^. Nonetheless, there were also researchers proving a rather reduced detrusor compliance in patients with CP^3,18^. It was reported that CP classified as GMFCS level IV and V (11/23, 47.83%) included patients with pathologic detrusor compliance in Bross’s previous study^18^, while only 11 (19.30%) children were classified as GMFCS level IV and V in this study. Besides, all these 11 non-ambulatory children possessed a good control over lower urinary tract according to their previous experiences confirmed by their caregivers, so the cases were proven to have a normal bladder compliance. Cause for the difference in results was still unclear, which might be related to the strict SDR indication for children with severe CP that CP with mental retardation would not be claimed to have SDR. However, further study is needed to clarify factors that could affect bladder compliance of patients suffering from CP.

Previous studies have shown the possibility of SDR causing bladder/bowel dysfunction after surgery^7^. Driven by concern about similar potential complication, pre-operative UDS has been conducted as a routine examination for all children with spastic CP in the Center since early 2020. Based on rhizotomy protocol applied in the SDR, it was necessary to judge all spinal nerve roots (rootlets) in cauda equina at L2 level with regard to their neurophysiological characteristics with trigger EMG in the surgery^6^. Rootlets should be classified as input and output ones. In addition, SAIR needs to be identified clearly in such a procedure, providing the potential for further investigation on the relations between trigger EMG patterns in channel of EAS when electrically stimulating those SAIR and UDS results in these patients. It was observed that the number of rootlets matching the SAIR criteria was around 16 to 18 in average in the cases. The single-level approach SDR via L2 made it difficult to determine which level these SAIR derived from during the surgery. Interestingly, it was found that in addition to those tiny sacral roots (rootlets), there were indeed many much thicker roots (rootlets) matching the SAIR criteria. Based on our experiences, these much thicker nerve roots were generally derived from level S1 or above. Nevertheless, given that these roots (rootlets) fit the criteria, they were still classified into the SAIR category for the investigation.

Trigger EMG data obtained in EAS during SDR rather than detrusor and external urethral sphincter was applied due to technical difficulties. As proposed by Sindou, the recording of EAS was easier to perform and cause less injury^25^. Thus, EAS neurophysiological exploration was extrapolated to striated urethral and detrusor in this research. The monitor of EAS might give information on the micturition function by extrapolation. This is because the striated urethral and striated anal sphincter was of the same somatic innervations, thus it could be reasonably postulated that such extrapolation might be logical^26^. In the study, the difference was minimized by adopting the average threshold value of all SAIR.

The intra-operative neurophysiological data unexpectedly showed that average threshold of those SAIR was higher in 12 patients (21.05%) with abnormal PVR (median: 0.86 mA vs. 0.46 mA), also indicating that in cases with increased PVR, a much higher input signal was needed to induce micturition reflex. One possible explanation was that during normal urination, the input signal gradually weakens until the completion of urination reflex after emptying as the bladder volume gradually decreased. In the process of urination in these children, when the remaining urine in the bladder was still in an abnormal amount, the reflex stopped, resulting in an abnormal amount of remaining urine. In addition, it was found that in 17 cases with constipation, the average threshold of SAIR was significantly higher than that of patients without constipation (median: 0.78 mA vs. 0.47 mA), which might also be attributed to the same mechanism.

Limitations of this study covered several aspects. Firstly, in addition to the absence of video fluoroscopy (limited device in our center), EMG data of pelvic floor could not be obtained in the current study. Difference still existed despite the overlap of neuronal circuits innervations between EAS and the bladder, bringing potential possibility to affect the accuracy of the conclusions. Nonetheless, correlation between trigger EMG data obtained during SDR and UDS results might provide a novel approach for researchers to investigate the mechanisms beneath those clinical presentations and UDS results. Secondly, the compound muscle action potential of EAS could hardly precisely reach at 20 μV during the SDR procedure. Thus, there might exist an error in the threshold of a certain SAIR. Considering that its average value was used for statistical analysis in the study, this type of error might be still within an acceptable range. Thirdly, the intelligence quotient in the patients was not assessed. In accordance with related articles, CP with lower IQ might present a worse bladder function^27^, which was not discussed in this study. Last but not least, only UDS was performed in patients who accepted SDR, making the results hard to represent the population of spastic CP. Nonetheless, it still offered the information of urinary dysfunction in part of these patients, thus raising the attention to the bladder function of patients suffering from spastic CP.

## Conclusion

Abnormal UDS results were prevalent in pediatric patients with spastic CP. Given its ability to detect silent bladder dysfunction, UDS becomes an important test in cases suffering from spastic CP, especially those with LUTS. Motor function level, age and average trigger EMG threshold of sphincter-associated input spinal nerve roots (rootlets) were closely related to abnormal UDS results (Supplementary information S1, S2).

## Supplementary Information

Supplementary Information 1.

Supplementary Information 2.

## Data Availability

The datasets used and analyzed during this study are available from the corresponding author on reasonable request.
