# Supplementary figures and images for: Urodynamic study findings and related influential factors in pediatric spastic cerebral palsy
Source: Sci Rep. 2022 Apr 28;12:6962. doi: 10.1038/s41598-022-11057-3 (PMC9050687; doi:10.1038/s41598-022-11057-3)

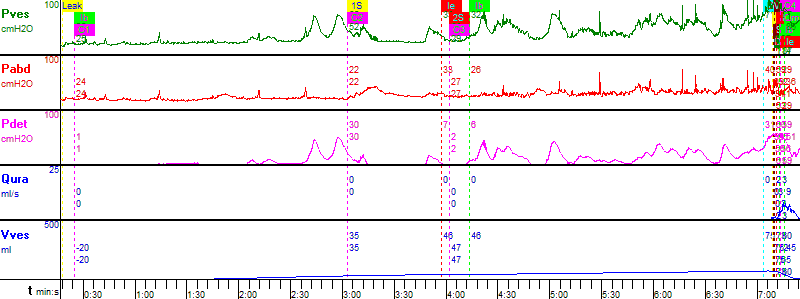


**
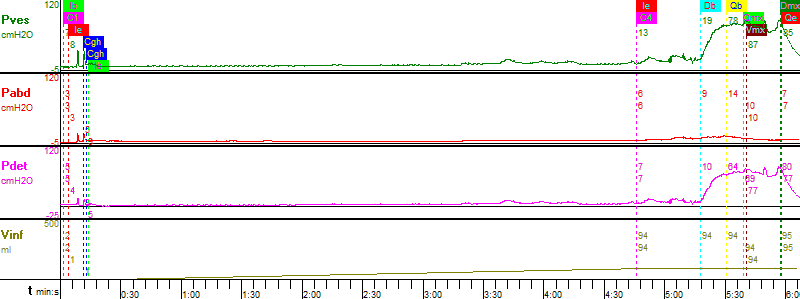
**

**
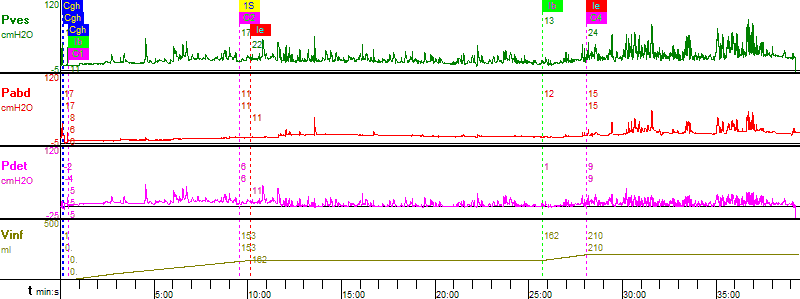
**

**
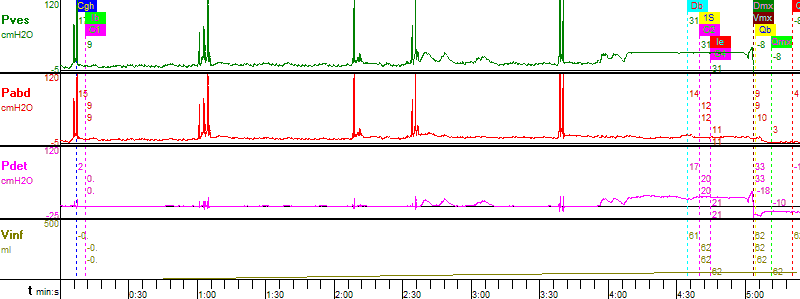
**

**
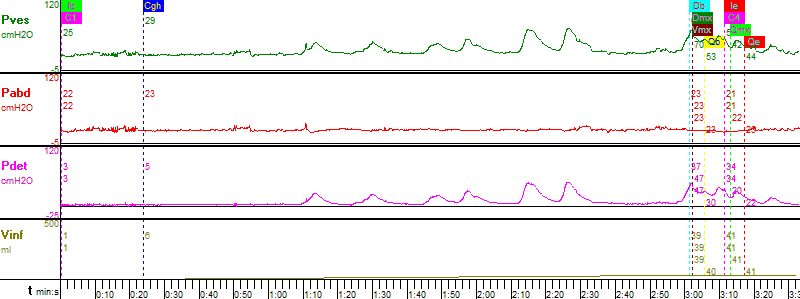
**

**
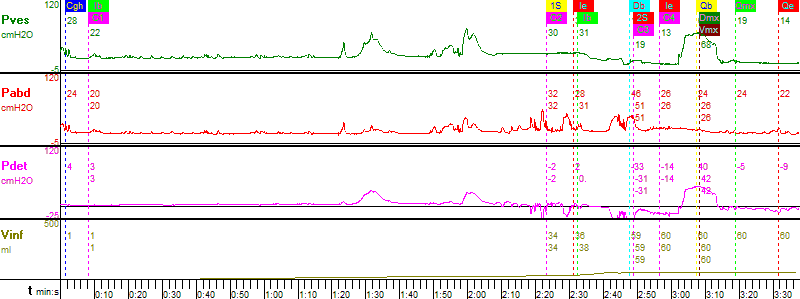
**

**
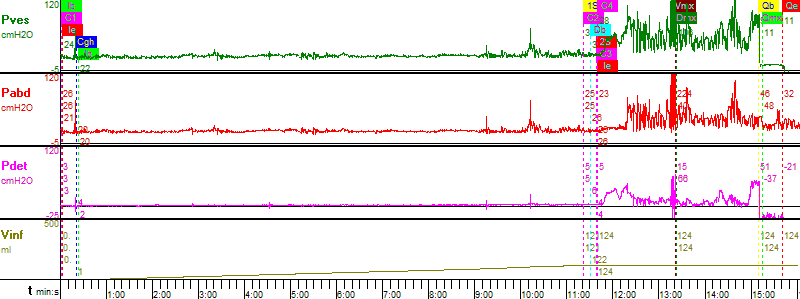
**

**
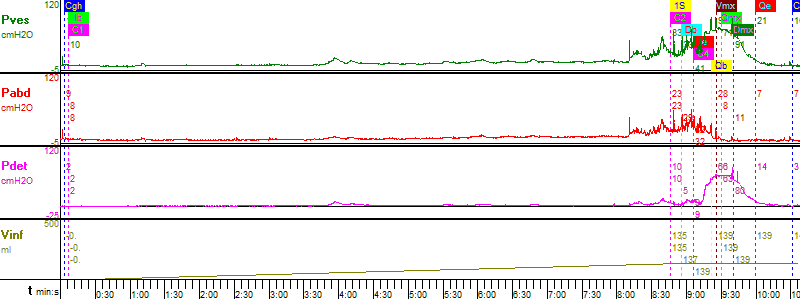
**

**
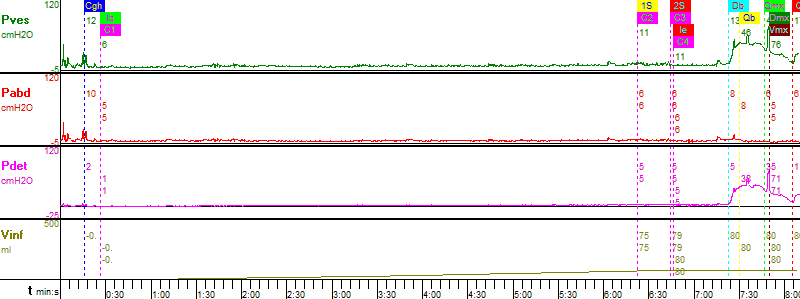
**

**
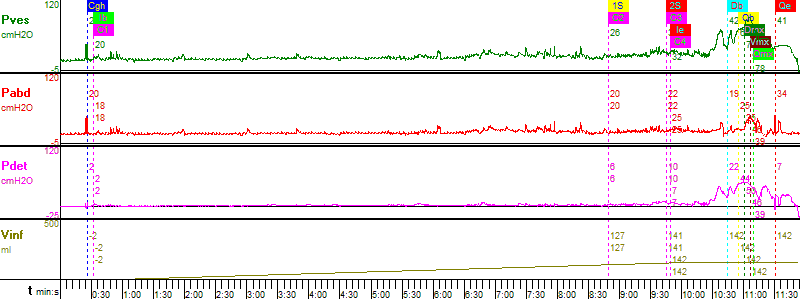
**

**
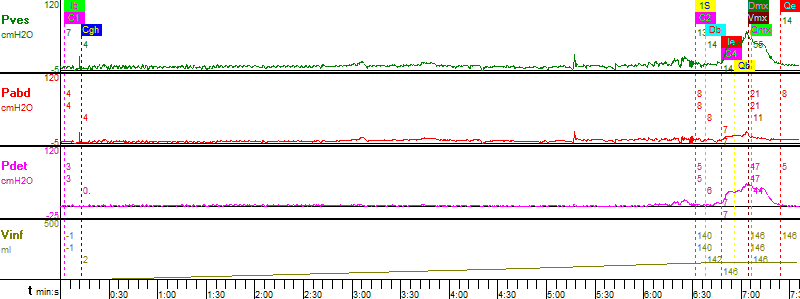
**

**
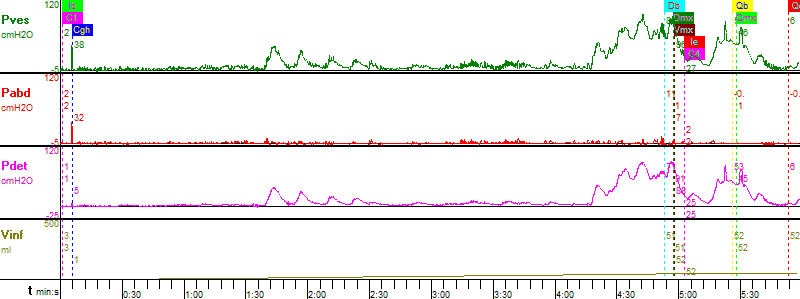
**

**
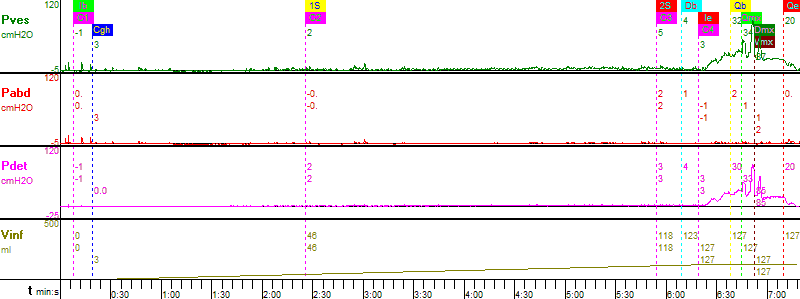
**

**
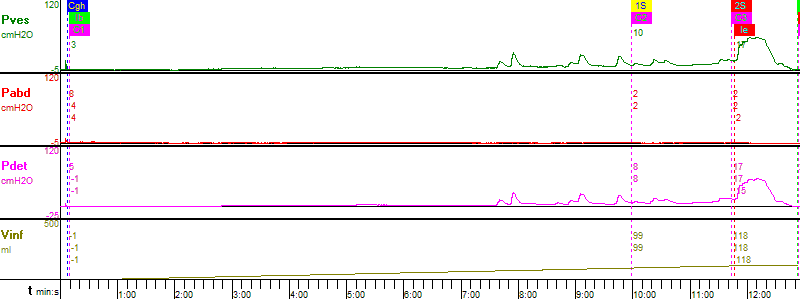
**

**
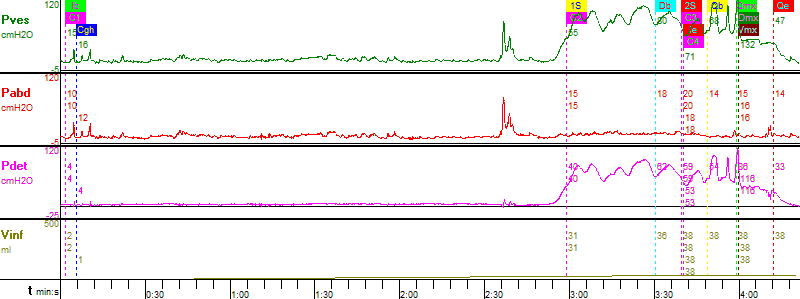
**

**
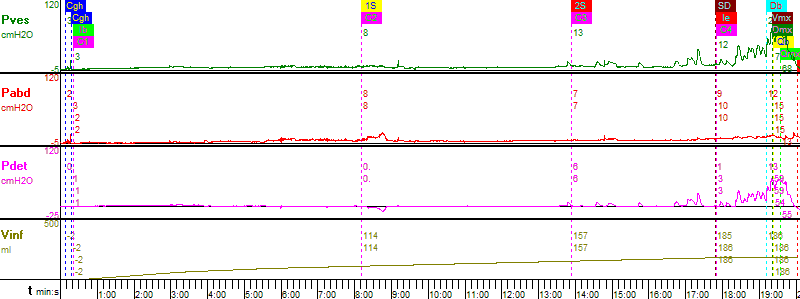
**

**
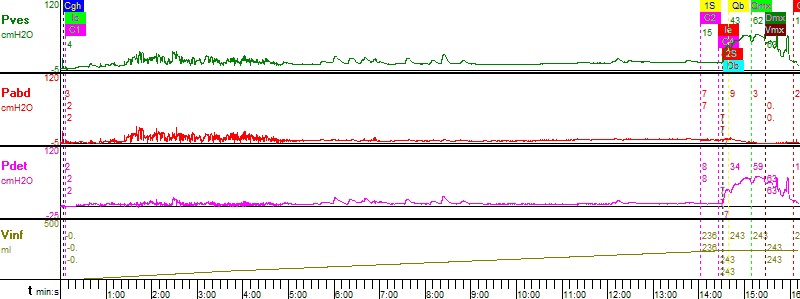
**

**
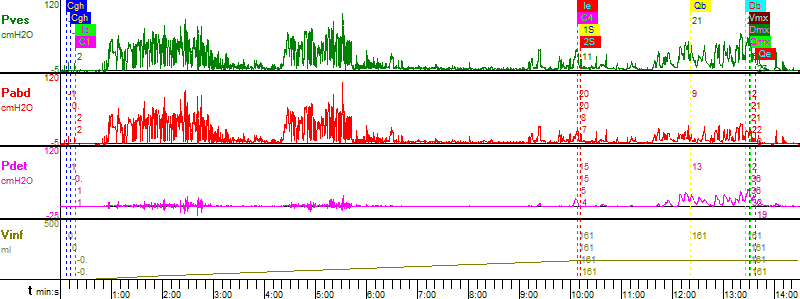
**


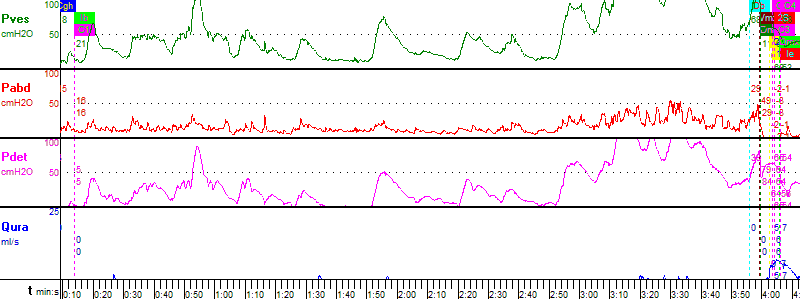


**
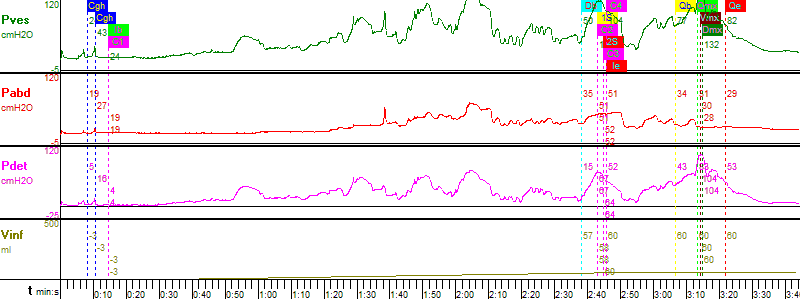
**

**
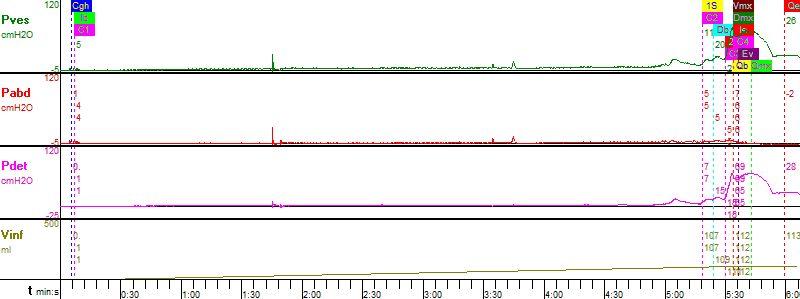
**

**
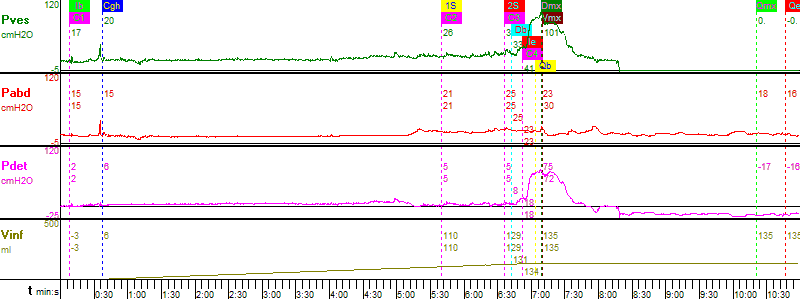
**

**
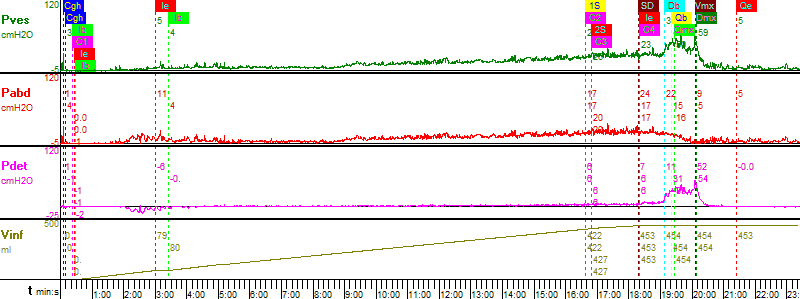
**

**
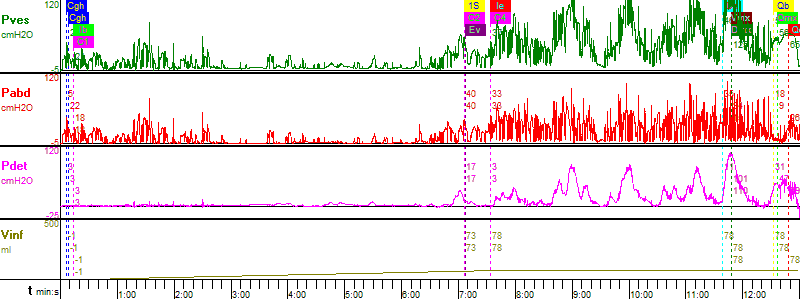
**

**
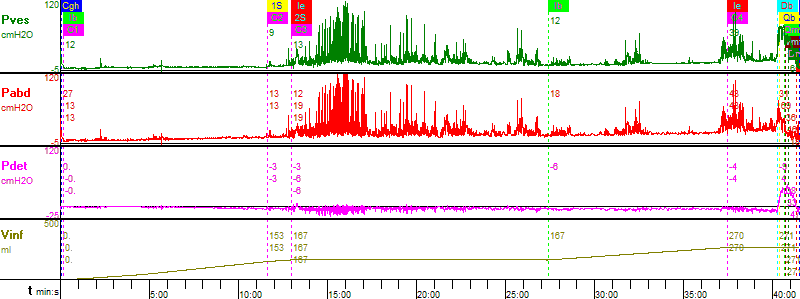
**

**
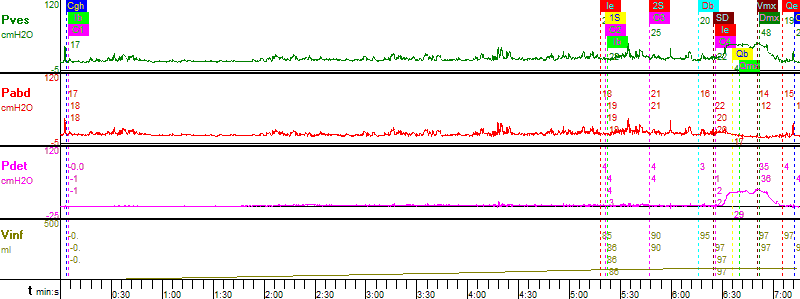
**


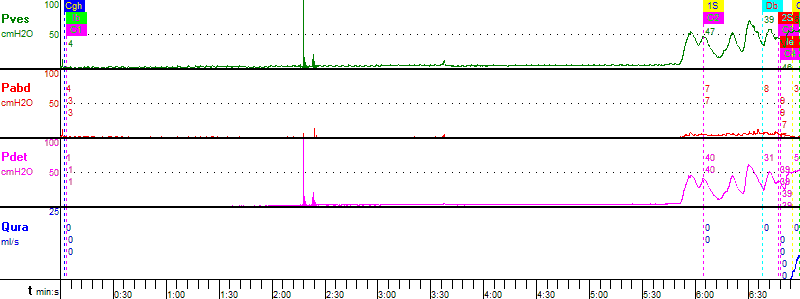


**
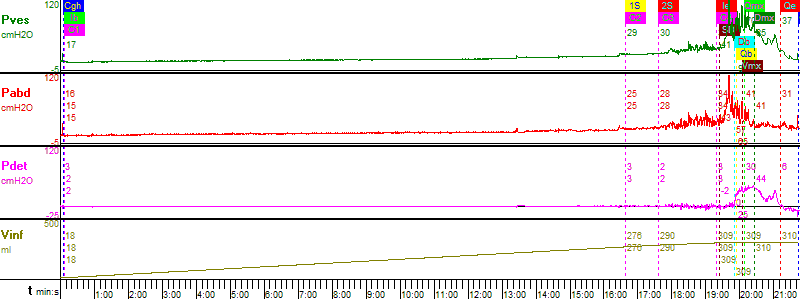
**

**
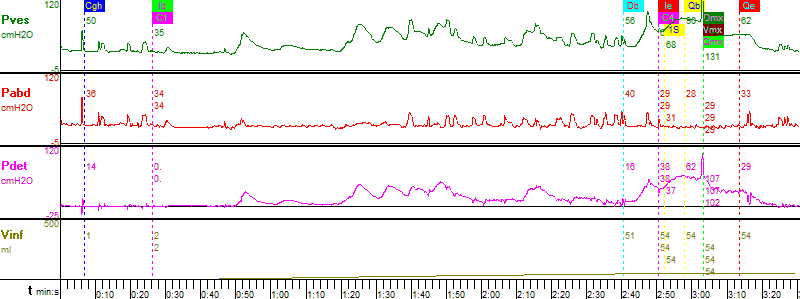
**

**
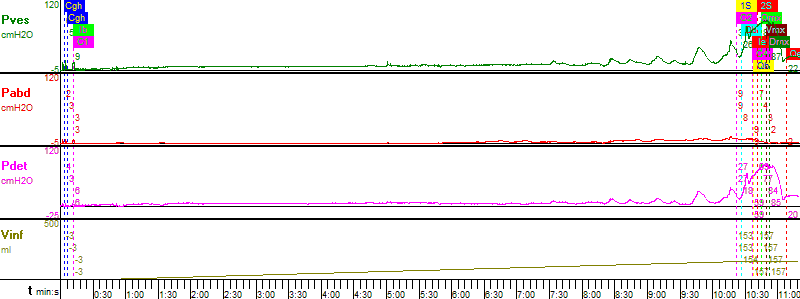
**

**
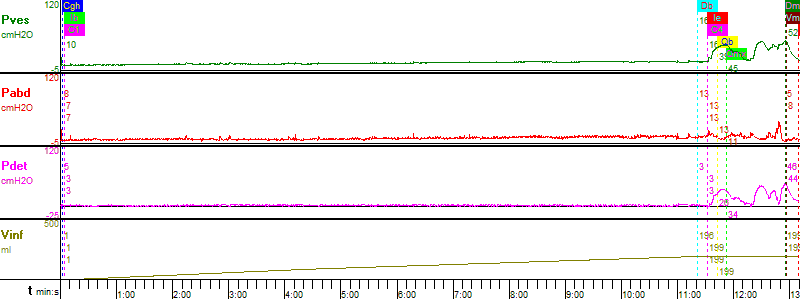
**

**
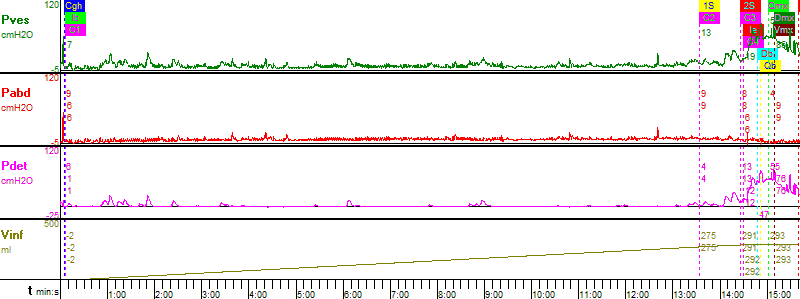
**

**
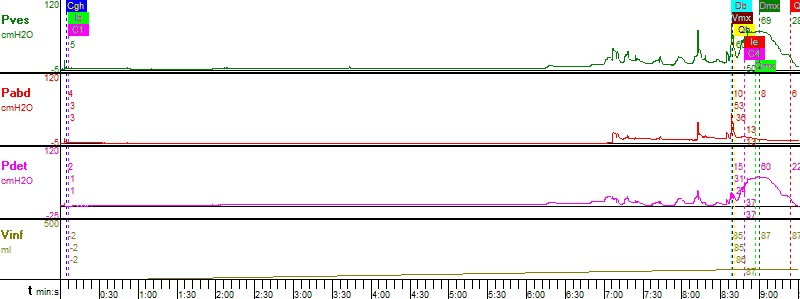
**

**
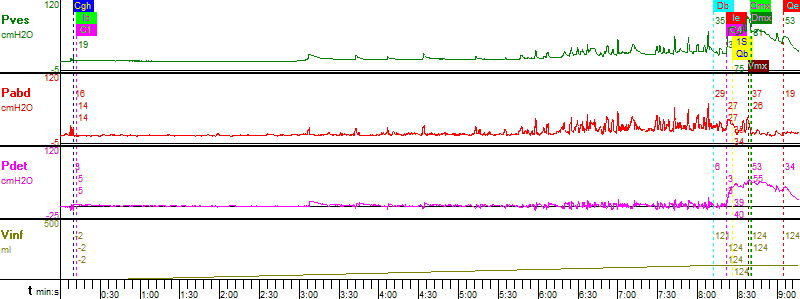
**

**
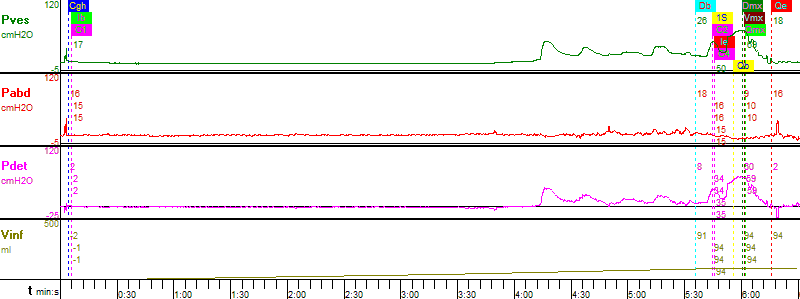
**

**
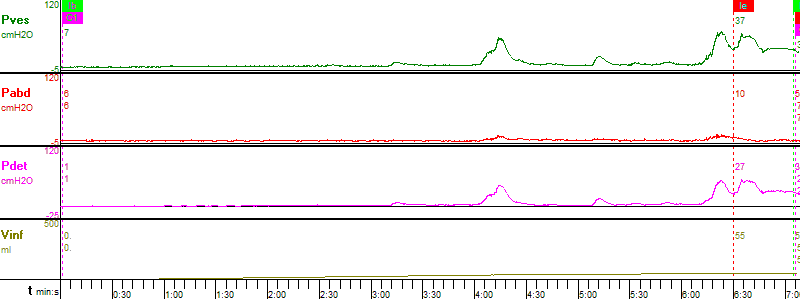
**

**
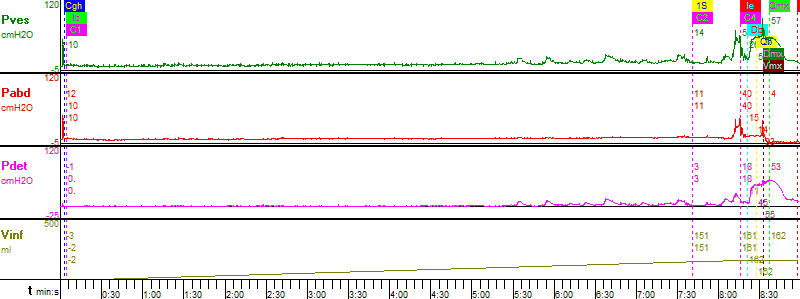
**

**
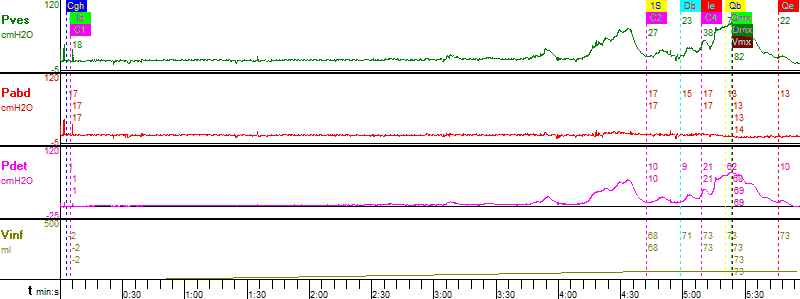
**

**
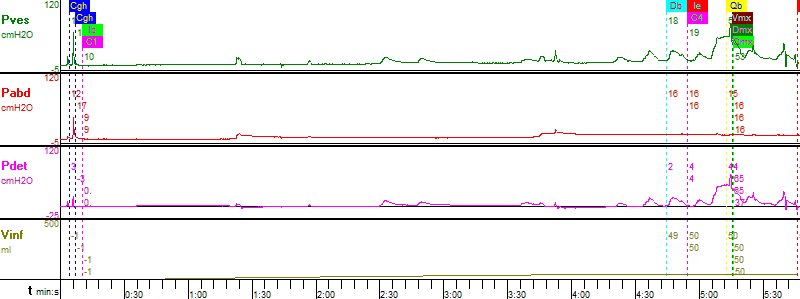
**

**
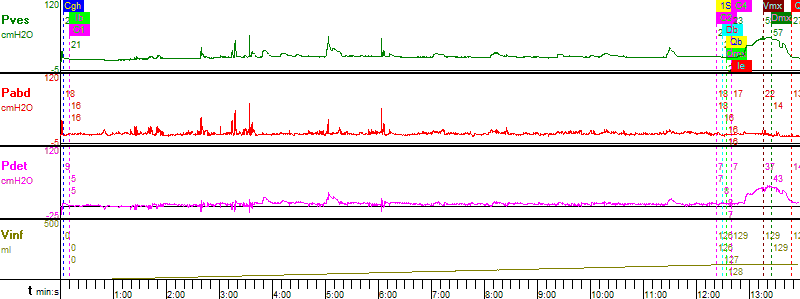
**

**
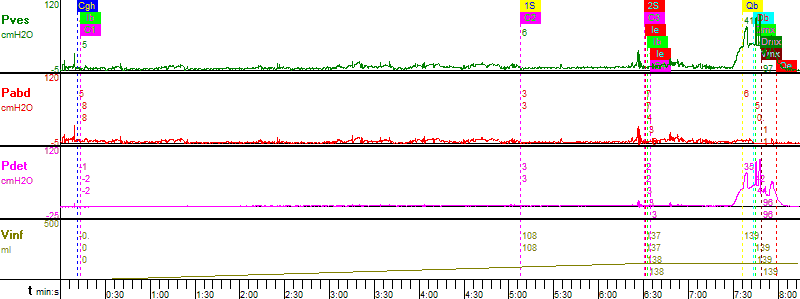
**

**
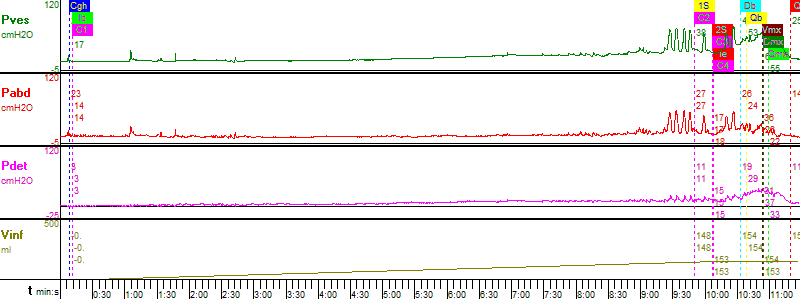
**


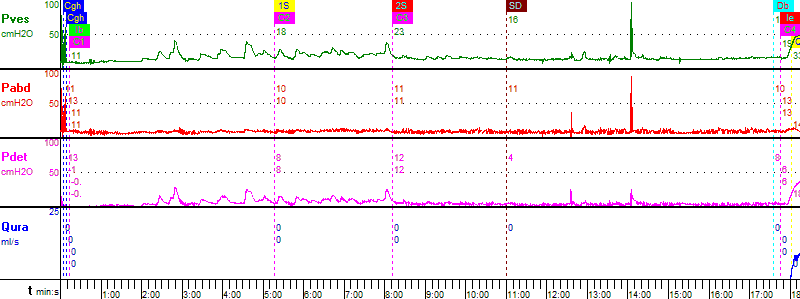


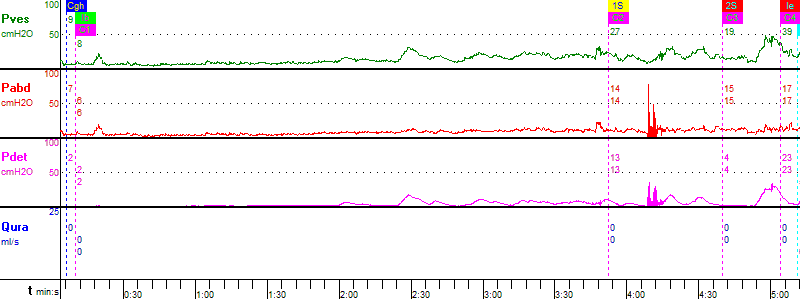


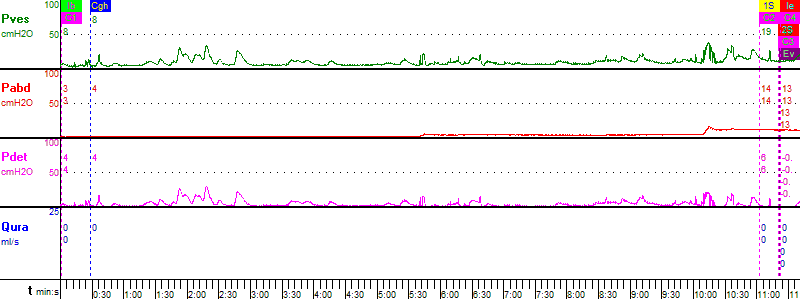


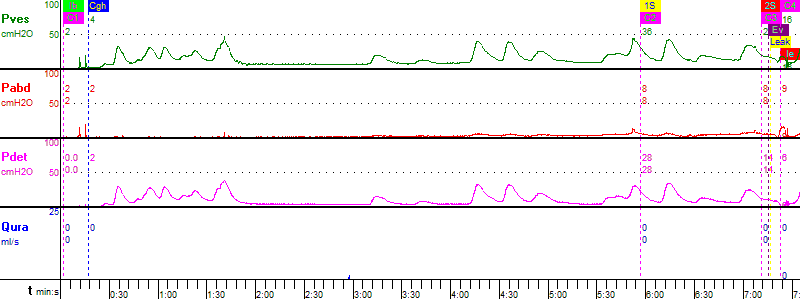


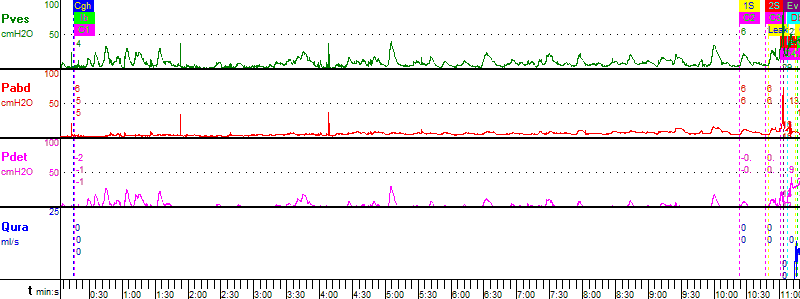


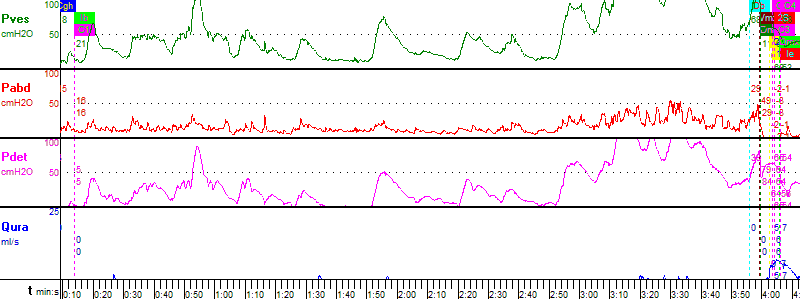


**
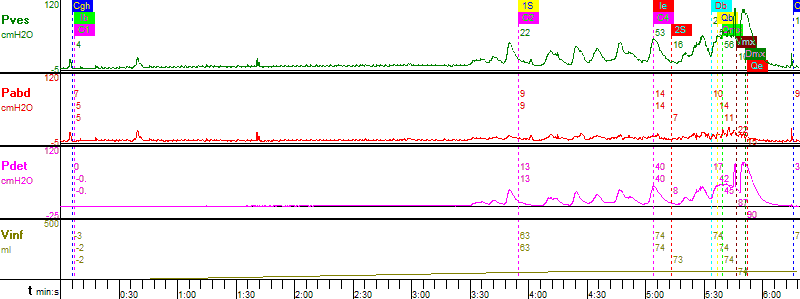
**

**
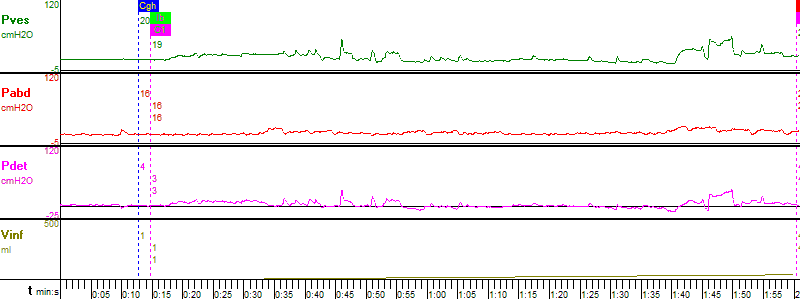
**

**
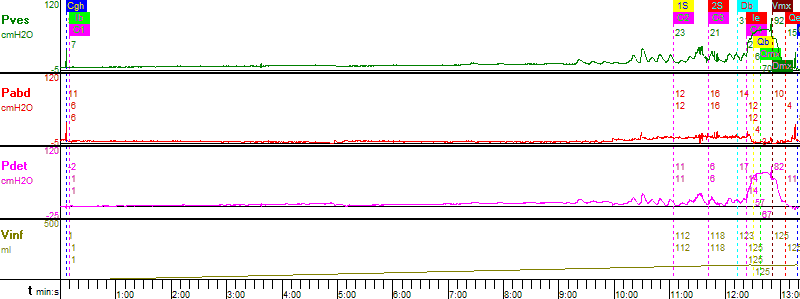
**

**
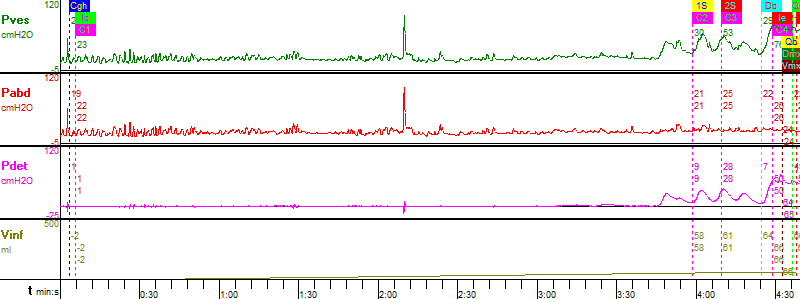
**


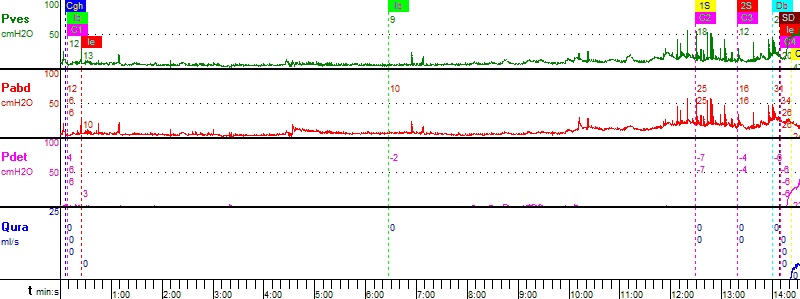


**
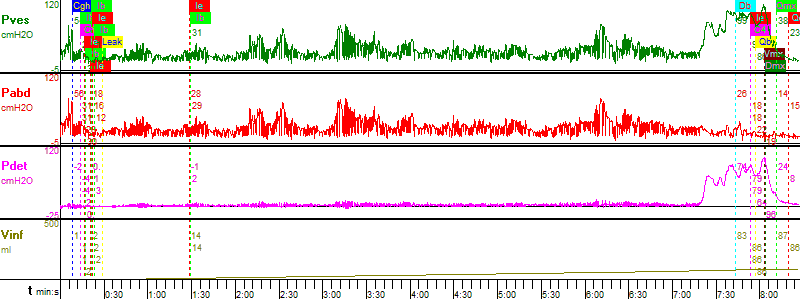
**

**
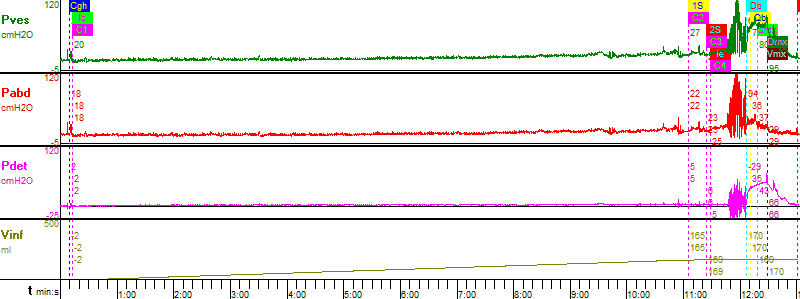
**

**
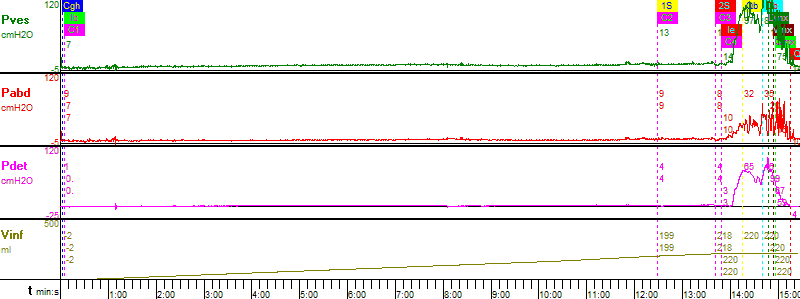
**


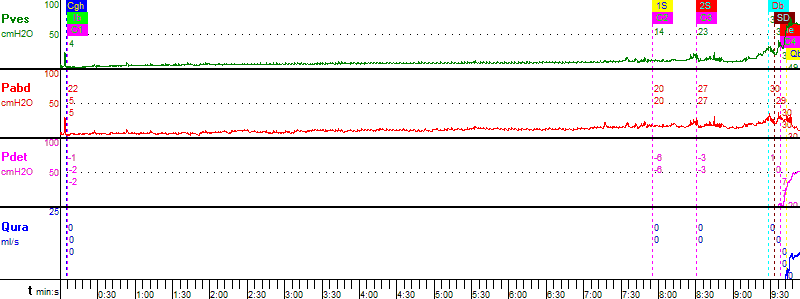

Supplement: Supplementary file 2 — Supplementary Information 2. [file 41598_2022_11057_MOESM2_ESM.docx]
